# A Self-Harm Awareness Training Module for School Staff: Co-Design and User Testing Study

**DOI:** 10.2196/69309

**Published:** 2025-06-02

**Authors:** Anne-Marie Burn, Hayley Gains, Joanna K Anderson

**Affiliations:** 1 Department of Psychiatry University of Cambridge Cambridge United Kingdom

**Keywords:** self-harm, schools, young people, youth, school staff, training, co-design, qualitative

## Abstract

**Background:**

The increasing prevalence of self-harm among adolescents is a significant public health concern. School staff are often the first professionals to notice when a young person is self-harming and are in a unique position to intervene and offer support. However, research indicates that many school staff members feel ill-equipped and lack confidence in how to respond. Negative or dismissive responses may discourage young people from seeking further help. There is an urgent need for targeted training interventions to equip school staff with the skills and knowledge necessary to support students who self-harm.

**Objective:**

This study aimed to co-design a self-harm awareness e-learning module for school staff in the United Kingdom.

**Methods:**

The e-learning module design and development was guided by a person-based approach over three participatory design cycles: (1) co-design sessions with experts in mental health, self-harm, and school-based training; (2) workshops with school staff to co-design the e-learning module components and explore their views on supporting students who self-harm; and (3) user testing of the prototype and focus groups with school staff to explore acceptability and feasibility. Data were thematically analyzed using the framework method.

**Results:**

Training content, videos, and quizzes were developed in collaboration with a panel of experts. Co-design workshops with school staff (n=11) informed the prototype module design, structure, and scripts for the training content and filmed scenarios, as well as highlighting potential barriers to and facilitators of implementation. User testing of the prototype with staff (n=20) yielded high usability ratings, demonstrating high levels of acceptability. Analysis of the qualitative user testing data generated four themes: (1) usability, (2) content and design, (3) feasibility, and (4) views on how the training improved knowledge and confidence.

**Conclusions:**

The Supportive Response to Self-Harm e-learning module was developed to enhance school staff’s knowledge and confidence in responding to self-harm. It was created with a user-centered design and a person-based approach and underpinned by psychological theory. Initial findings indicate that the training is acceptable and feasible. Further research will involve a mixed methods pilot feasibility study to assess the effectiveness of the program. This will provide the necessary evidence for a large-scale rollout in schools.

## Introduction

### Self-Harm Among Adolescents

In the United Kingdom, self-harm is defined as “all acts of intentional self-poisoning or self-injury, irrespective of the apparent purpose” [1]. Self-harm has been widely researched as a feature of clinical populations, including those with a diagnosis of borderline personality disorder, bipolar disorder, and posttraumatic stress disorder. However, self-harm is increasingly prevalent among adolescents in the general population [2]. A survey of individuals aged 15 years in England found that 34% had deliberately hurt themselves at some point in their lifetime, an increase from 22% in 2014 and 25% in 2018 [3]. Between 10% and 20% of young people who self-harm present to hospital, whereas many other individuals sustain injuries that are classified as low risk and, therefore, do not receive hospital care [4]. Despite this, low-level self-harm behaviors are indicators of psychological distress and may require professional intervention. Young people who self-harm are at a high risk of adverse outcomes, including poor mental health in adulthood and an increased risk of suicide [5,6]. Research indicates that self-harm can be a persistent behavior. In one study, 19% of those who self-harmed in adolescence were still self-harming 10 years later [7]. This underscores the need for early detection and timely interventions to prevent long-term negative outcomes in young people who self-harm.

### Self-Harm in the School Setting

The UK government’s green paper highlights the essential role of schools in supporting young people’s mental health and providing earlier access to support [8]. Self-harm in school settings often stems from various factors, including bullying, anxiety, and the stress associated with academic pressures, and, in some cases, might serve as an attempt to seek acceptance within a peer group [9]. A recent study identified bullying, adults not listening, being picked on by a teacher, and racism as risk factors for self-harm in school settings [10]. Despite high prevalence, there is a notable absence of policies in the United Kingdom that outline how schools should address and support students who self-harm. Consequently, it becomes the responsibility of each school to navigate this challenging issue and establish their own protocols and practices for intervention and support.

School staff are often the first adults to notice when a young person is self-harming and are in a key position to intervene and provide timely support. However, research has shown that many staff members feel ill-equipped, lacking the knowledge, confidence, and resources to effectively support students [11,12]. While many staff members are willing to help, they report a need for training to improve their knowledge and confidence. A survey of UK teachers found that awareness of self-harm was inconsistent and their responses often included shock, panic, and anxiety [13]. Such reactions may discourage young people from seeking help and urge them to conceal their behavior [14]. There is evidence suggesting that most young people who self-harm do not seek support due to concerns about receiving a negative response from those they confide in [14-16]. Furthermore, the prevalence and the impact of self-harm is often underestimated in schools and not prioritized in the curriculum despite students expressing a need for information on the topic [9].

### Self-Harm Training for School Staff

Training interventions could be an effective way to increase school staff’s knowledge and confidence in responding to self-harm. However, there is a lack of rigorously evaluated training programs for school staff to support students who self-harm [17]. A UK survey found that 52% of staff members recruited from 222 schools reported having received some training about self-harm, but only 22% rated the training as adequate [18]. In a smaller study of 30 teachers, 5 had encountered a student who self-harmed, but none had received any self-harm training, and all expressed a need for supervision and increasing their knowledge [19]. Self-harm training for pastoral staff has been shown to provide a safe, reflective space where staff can better understand self-harm, address their own anxieties about the topic, and promote a shift in their attitudes and beliefs [20].

Consequently, there is a significant gap in accessible and feasible training programs for schools to effectively address student self-harm. Researchers in the United Kingdom developed a 30-minute e-learning module on self-harm for teachers [21]. The module was created with input from a head teacher and 2 mental health professionals, but limited information is available on the content or the development process. Teachers’ actual knowledge of self-harm measured using the Self-Injury Knowledge Questionnaire [22], significantly increased immediately after the intervention (*z*=9.62; *P*<.001; *r*=0.73; N=167 teachers). Perceived knowledge and confidence in addressing self-harm and speaking to young people about self-harm also showed significant improvement. However, the sustainability of these outcomes is not known. Acceptability ratings were high, with 90.7% of teachers reporting that e-learning was a good method for receiving training and that they found the training both accessible and engaging. However, as the training was developed specifically for teachers, the study does not provide evidence on the feasibility and acceptability of self-harm e-learning among nonteaching school staff, whose involvement is essential for a whole-school approach to prevention and intervention.

### This Study

The aim of this study was to co-design a stand-alone e-learning training module to equip school staff with the skills to communicate supportively with students about self-harm, respond appropriately to disclosures or incidents, and encourage students to seek help. This training intervention builds on the existing Supportive Response to Self-Harm (SORTS) web-based training program and resource toolkit as part of a whole-school approach [23-25]. This previous work has highlighted the need for an e-learning module incorporating scenarios and conversation starters, which staff members could complete as part of their induction or continuing professional development (CPD) training.

This paper describes the design and development of the SORTS e-learning module using a person-based approach as a framework [26]. The training intervention was theoretically informed and guided by three principles from existing research evidence to (1) raise awareness and understanding of self-harm among school staff, (2) improve staff confidence when responding to self-harm, and (3) encourage a whole-school approach to self-harm. Using a participatory development cycle, we co-designed the e-learning module components with an expert panel and potential end users (school staff members) to inform a rapid prototype, followed by user testing to assess its usability, acceptability, and feasibility.

## Methods

### Ethical Considerations

Ethics approval was granted by the University of Cambridge Department of Psychology Research Ethics Committee (reference PRE.2024.006). All participants were fully informed about the project, including the limits on confidentiality and their right to withdraw from the study at any time without giving a reason. They were informed that personal data would be kept confidential and managed in accordance with the Data Protection Act, the Research Governance Framework for Health and Social Care, and the conditions of Research Ethics Committee review. Electronic consent was obtained from each participant before taking part. All audio recordings of the focus groups were taken on an encrypted voice recorder and transcribed by a university-approved transcription company. Transcripts were deidentified and stored electronically in separate, password-protected folders that only the research team had access to. All participants were offered a £40 (US $51.93) online shopping voucher for their time. Reporting followed the COREQ (Consolidated Criteria for Reporting Qualitative Research) guidelines.

### Underlying Theory and Logic Model

The theoretical underpinning of the SORTS program is the self-efficacy theory by Bandura and Adams [27], which describes self-efficacy as one’s beliefs about their capabilities to plan and execute actions that are required to produce a desired outcome. Studies show that teachers with high self-efficacy are more confident and effective in their teaching and classroom management. Students also view these teachers as more competent and trustworthy [28]. Teachers’ attitudes and confidence to support students’ mental health may be determined by their knowledge, interests, experience, and self-efficacy [29]. The theory of change hypothesis is that completing the SORTS training program will result in school staff’s improved knowledge about self-harm and increased confidence when responding to students who self-harm, which in turn will facilitate student help seeking and earlier access to support. The theory of change model (Multimedia Appendix 1) will be revised iteratively as new findings emerge.

### Research Plan

We were able to draw on a previous systematic review and focus groups with young people and school staff to create the e-learning content [17,23,25]. Following Medical Research Council guidance, the e-learning module development involved appropriate users at all stages of the process [30]. In addition, the study used a person-based approach, which actively involves potential end users in intervention planning and leads to the development of guiding principles [26].

**Figure 1:**
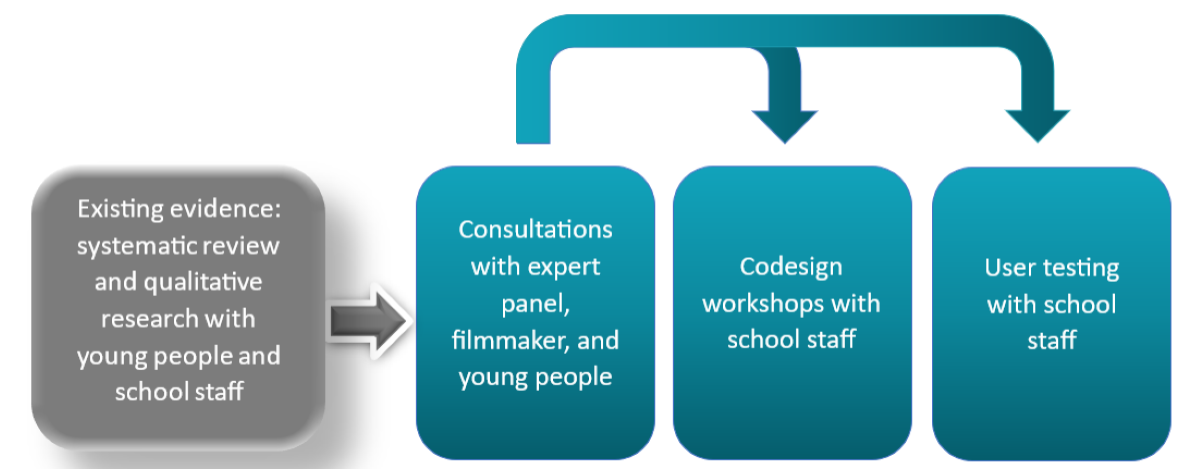
Research process for the design and content of the e-learning module.

The research and development cycle included three participatory design phases: (1) consultation with an expert panel, (2) co-design workshops, and (3) user testing (the process is outlined in Figure 1). This iterative approach enabled us to assess the utility, acceptability, and feasibility of the training intervention and incorporate feedback into the design and development. At the start of the study, we invited public collaborators with a range of perspectives and roles to join an “expert” public involvement panel (subject matter experts). Qualitative co-design workshops were conducted with school staff to refine and finalize the training scenarios, gather feedback on the user interface design, and capture potential users’ views on prospective acceptability and feasibility [31]. Findings from the co-design workshops informed the design and development of the prototype e-learning module. We commissioned a technical developer with an established record of creating web-based training for the English National Health Service on mental health topics. In addition, we commissioned a filmmaker who specializes in creating films about children and young people’s mental health to create two filmed scenarios (1) depicting a staff member discovering a student’s self-harm and (2) showing a poor response to a student’s disclosure compared to a more supportive response. In the user testing phase, the prototype e-learning module was given to a sample audience to identify any usability issues, acceptability, and feasibility [32] and measure changes in knowledge and confidence.

### Procedures

#### Initial Design and Development of the e-Learning Module With Subject Matter Experts

We held a series of co-design meetings with a panel of subject matter experts to advise and develop ideas for the e-learning module training content and web elements. Panel members had expertise in young people’s mental health and experience working in school settings. They included 2 senior mental health school staff members, 2 well-being leads, a teaching assistant, an intervention teacher, and a behavioral support officer. In addition, the panel included 2 self-harm trainers from a UK mental health charity that delivers self-harm awareness training for schools. Experts provided valuable insight on the training needs of school staff, the appropriate level of information about self-harm, and the challenges of managing self-harm in the school context. Co-design sessions focused on designs of mock-ups of the user interface, a wireframe structure of the module, types of questions for the interactive quizzes, draft video scripts, and storyboards depicting school-based scenarios of student-staff interactions regarding self-harm. Scenarios enable the learner to think about real-life situations as well as creating a more immersive and engaging learning environment. We worked with a filmmaker with expertise in producing mental health–related content to further refine the scripts for the filmed scenarios. We then met with a group of 3 young people (aged 18 years) to obtain their perspectives to ensure that the scripts were realistic depictions of a pupil-teacher interaction.

#### Participant Recruitment for Staff Workshops and User Testing

For both data collection phases, we aimed to recruit a range of school staff members working in different roles (eg, student support, administration, teaching assistants, and teachers). As far as possible, we purposefully sampled staff to ensure that a diversity of backgrounds was represented. In total, 4 state-maintained secondary schools and colleges took part in the study. We recruited schools via the team’s local networks in the South and East of England. One school was situated in an urban area (London), and 3 schools were within areas that have been identified as “populations in focus” by the National Institute for Health and Care Research Applied Research Collaboration East of England due to having high health needs [33]. An email detailing information about the study, research activities, and potential involvement was sent to a key contact person (eg, mental health lead) within each school. The school contact person helped identify and approach potential participants and raise awareness of the study by distributing study information to school staff. School staff members who expressed an interest in participating were asked to contact a study researcher for further details, who then sent a participant information sheet and a consent form. Informed consent was obtained from each participant before taking part. Participants also completed a demographics form and received thank-you vouchers for their time.

#### Co-design Workshops With School Staff

**Figure 2:**
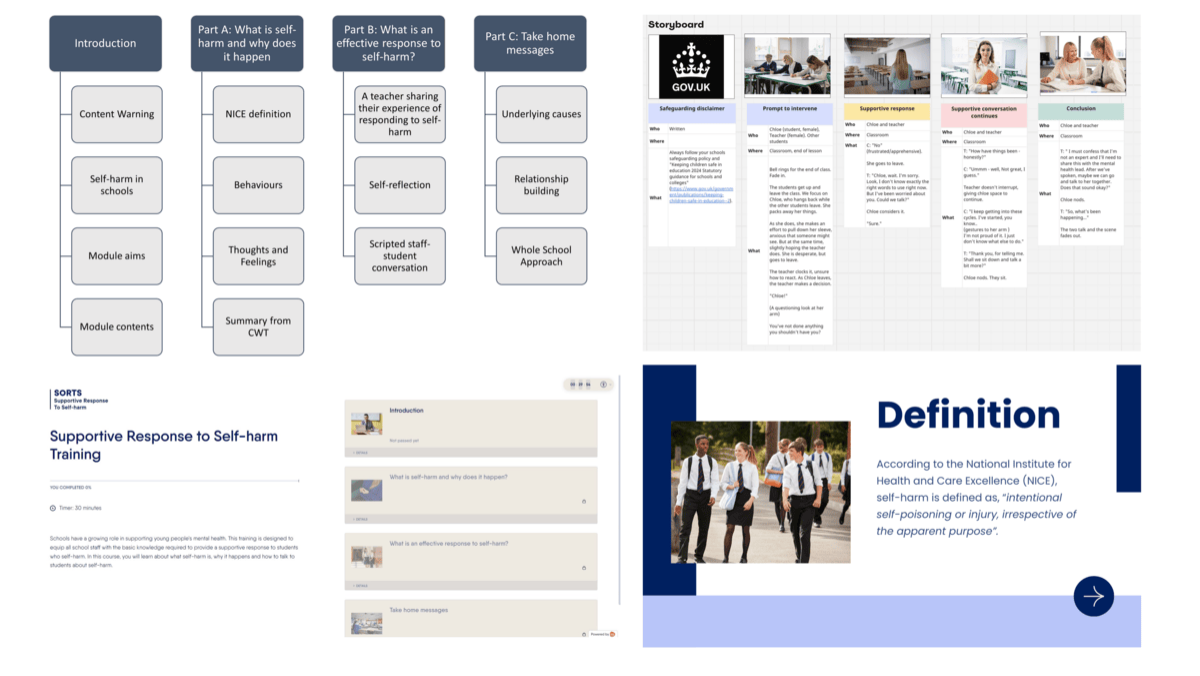
Example materials used in the co-design workshops with school staff. Note: images with visible people are stock images sourced from iStock.

A semistructured topic guide was developed for the staff workshops (Multimedia Appendix 2). Participants were asked to complete a preworkshop task to familiarize themselves with the e-learning training content and designs and note any other knowledge areas, skills, and techniques that they would like to incorporate into the training. We then ran in-person workshops at 2 schools to obtain their initial feedback on the proposed designs and generate new ideas on the content and training scenarios. Sessions lasted up to 2 hours and were facilitated by an experienced qualitative researcher (AMB) with support from another member of the research team (HG). Materials were presented to participants, including the initial wireframe structure, visual design concepts, mock-ups of the module, interactive quizzes, and scenarios that had been developed with the expert panel (Figure 2).

#### User Testing of the e-Learning Module

**Figure 3:**
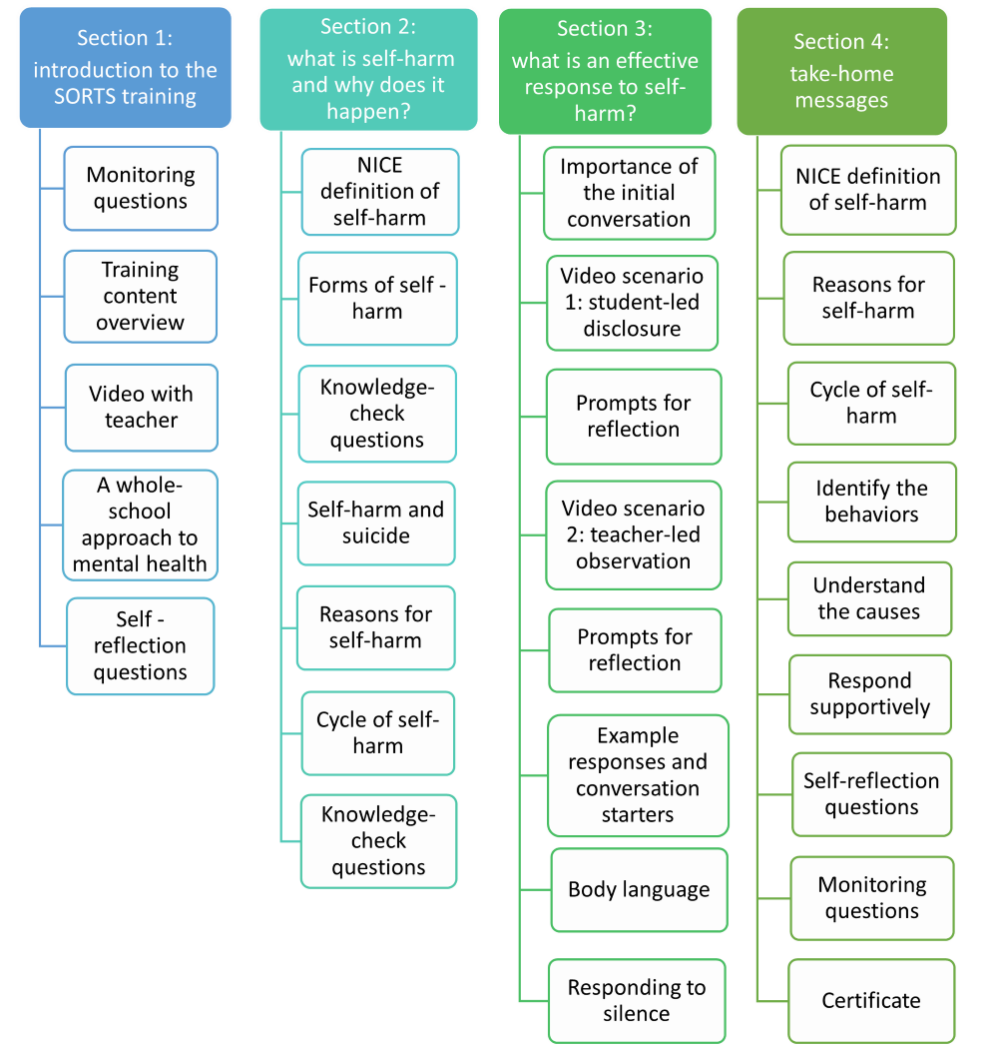
Final structure of the e-learning module for user testing. NICE: National Institute for Health and Care Excellence; SORTS: Supportive Response to Self-Harm.

**Figure 4:**
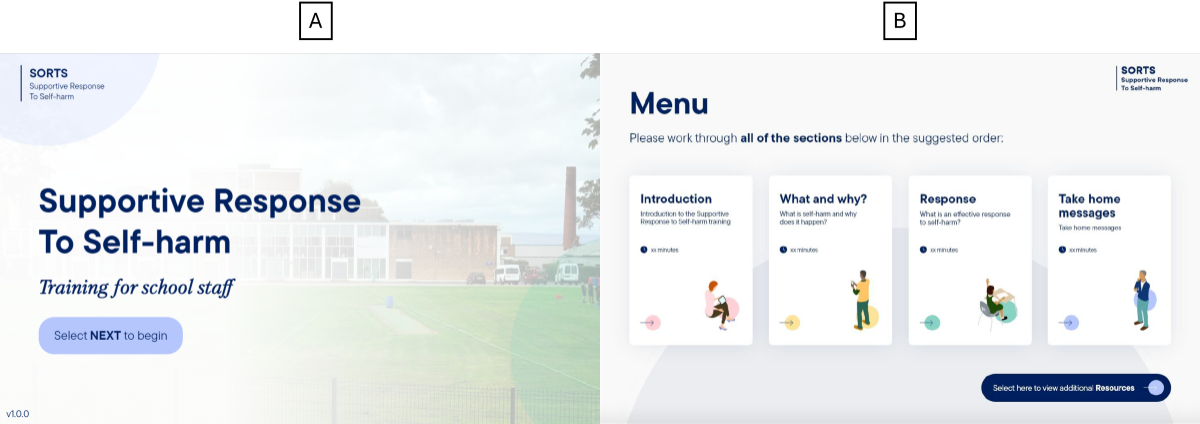
Supportive Response to Self-Harm (SORTS) e-learning module—welcome screen (A) and menu screen (B) for user testing.

**Figure 5:**
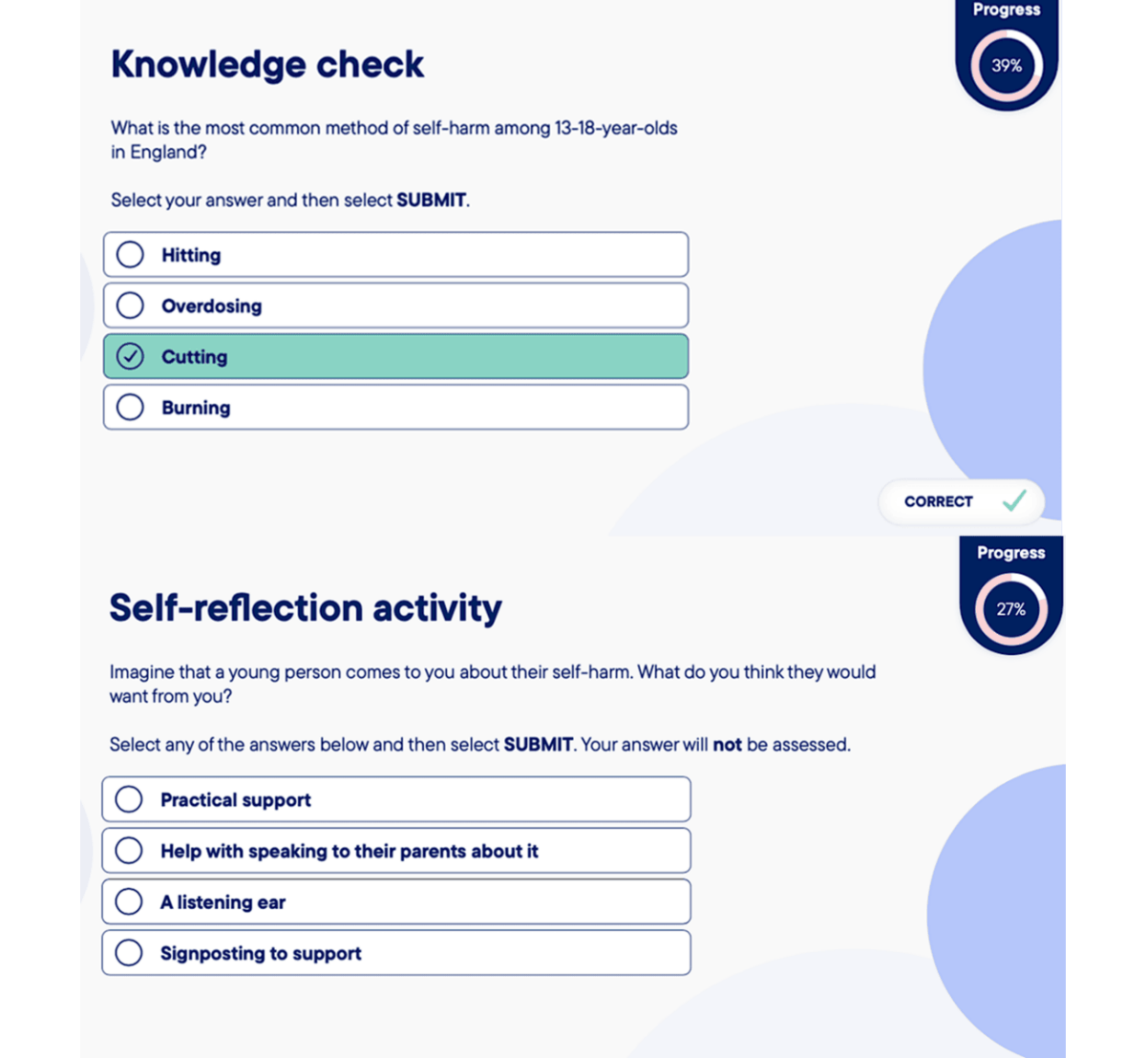
Examples of quiz questions in the e-learning module.

**Figure 6:**
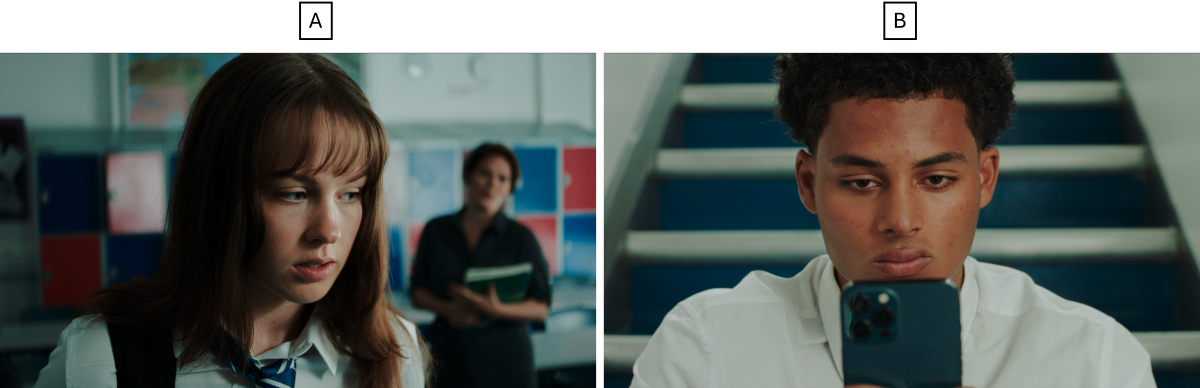
Screenshots of filmed scenarios—Chloe’s story (A) and Jack’s story (B). Note: images of actors from the film are intellectual property of the authors.

Once the e-learning prototype was finalized, we conducted a user testing phase. Participants were given 2 weeks to complete the 30-minute e-learning module and a short survey to capture metrics on usability (Multimedia Appendix 3). A topic guide was developed to explore usability and acceptability, as well their perspectives on changes in their knowledge and confidence (Multimedia Appendix 4). Follow-up focus groups were conducted at 3 schools; one of these schools had taken part in the previous workshops, but new staff members were recruited for the user testing. Focus group sessions lasted approximately 60 to 70 minutes. During the sessions, we explored staff’s views on the design, usability, and navigability of the module, and similarly to the previous design and development phase, participants were asked about their views on the acceptability, feasibility, and barriers to implementation. The finalized module structure is shown in Figure 3. Visuals of the e-learning module prototype are shown in Figures 4 and 5, and screenshots from the filmed scenarios are shown in Figure 6.

### Analysis

All focus groups and workshops were recorded, transcribed professionally, thematically analyzed using the framework method [34], and triangulated with usability data. The transcripts were checked for accuracy, and all identifying information was removed before being entered into NVivo (version 12; QSR International) for data management. A detailed analysis plan was drawn up to guide members of the research team through the analysis. The process comprised the following stages: transcription; familiarization with the transcripts; coding; and development of a working analytical framework, application of the framework, charting of data into the framework matrix, and interpretation of the data. The team (AMB, HG, and JKA) began by familiarizing themselves with a subset of transcripts from each participant group and using a combination of deductive and inductive coding. Team members met to discuss how the codes could be grouped into categories, and these formed 2 initial working analytical frameworks (one for student data and one for school staff data). The working analytical frameworks were entered into NVivo and applied to all transcripts. Once all the transcripts had been coded, Microsoft Excel framework matrices were generated, which enabled the data to be charted by AMB and HG This involved creating detailed summaries in each cell of the matrix while retaining key verbatim quotes. The team met regularly to discuss potential themes and interpret the data. Monitoring data were exported from the SORTS e-learning module and analyzed using R Statistical Software (version 4.3.1; R Foundation for Statistical Computing). Two-tailed, paired samples *t* tests were conducted to assess whether there had been a significant change in participants’ self-reported understanding and confidence rating at the beginning and end of the module.

## Results

This section provides the results from the co-design workshops and user testing phase. Themes from the qualitative analysis are illustrated with example quotes from school staff.

### Participants in the Workshops and User Testing

In total, 31 school staff members (n=23, 74% female; n=8, 26% male) participated in the study from 4 schools (n=3, 75% mixed sex and n=1, 25% single-sex boys’ school). The single-sex boys’ school took part in both phases, but different staff members attended the sessions. Participants’ years of experience were 0 to 5 (21/31, 68%), 5 to 10 (4/31, 14%), 15 to 20 (3/31, 9%), and ≥20 (3/31, 9%), and they had a range of roles (Table 1).

**Table 1:** Roles of school staff members who participated in the co-design workshops and user testing (N=31).

Role		Participants, n (%)	Previous mental health training
**Co-design workshop 1 (n=5)**			
	Associate assistant head (SLT^a^)	1 (20)	No
	Deputy SENCO^b^	1 (20)	Yes
	Head of department for art and design	1 (20)	No
	TA^c^ and food technician	1 (20)	No
	Safeguarding officer	1 (20)	Yes
**Co-design workshop 2 (n=6)**			
	Deputy head (SLT)	1 (17)	No
	Head of humanities (SLT)	1 (17)	Yes
	School chaplain	1 (17)	No
	Pastoral lead	1 (17)	Yes
	Assistant principal and DSL^d^ (SLT)	1 (17)	Yes
	Administrator (secretary)	1 (17)	No
**User testing in school A (n=9)**			
	Assistant head teacher (SLT)	1 (11)	Yes
	Higher-level TA	1 (11)	Yes
	Head of drama	1 (11)	No
	Teachers (English and social sciences)	2 (22)	No
	Career and marketing coordinator	1 (11)	No
	Head of geography	1 (11)	No
	Post 16 support officer	1 (11)	Yes
	Librarian	1 (11)	No
**User testing in school B (n=6)**			
	Senior mental health lead	1 (17)	Yes
	Head of humanities	1 (17)	No
	Cover supervisor (previously TA)	1 (17)	Yes
	Head of maths	1 (17)	No
	Teacher of religious education	1 (17)	No
	Unit manager of the kitchen	1 (17)	No
**User testing in** **school C (n=5)**			
	Safeguarding officer	1 (20)	Yes
	Assistant head teacher and DSL	1 (20)	Yes
	Designated mental health lead	1 (20)	Yes
	Administrator (receptionist)	1 (20)	No
	Deputy SENCO	1 (20)	Yes

^a^SLT: senior leadership team.

^b^SENCO: special educational needs coordinator.

^c^TA: teaching assistant.

^d^DSL: designated safeguarding lead.

### Findings From Staff Co-Design Workshops

The qualitative analysis from the co-design workshops generated three main themes: (1) prospective acceptability of a self-harm awareness training module; (2) module design, structure, and content; and (3) potential barriers to and facilitators of implementation. This feedback was used to inform the design, development, and implementation of the training module.

#### Theme 1: Prospective Acceptability of a Self-Harm Awareness Training Module

Participants highlighted the need for guidance and training on how to manage self-harm, particularly because they worried about saying the wrong thing and inadvertently causing further distress for a student:

It’s [having] that confidence of going, “Right, what you’re going to say is then not going to make a negative impact to that child five hours later at home.” And not having that fear of going—if you say the wrong thing, this might then make them progress even worse.

Head of drama

Some staff members noted that there were some practical subjects, such as food technology and art, where they had seen students use objects to self-harm and they had not received any training in how to manage that:

It just needs to be addressed. Because I’ve taught for a really long time and, as an art teacher and probably in food, there’s a lot of objects where students can self-harm. And it’s a practical subject, so I have seen students self-harming in lessons, I have seen students going to self-harm, or I notice things. I have never had any formal training on that.

Associate assistant head

Staff welcomed the idea of a training module but expressed concerns about being overwhelmed by the content and the potential for it to be distressing for some staff members. They felt that it was important for the training module to be sensitive to the lived experiences of school staff as they may have encountered self-harm both personally and professionally:

Depends on your own personal circumstances, as well, as to how you feel you can answer the questions. Because some people are more personally affected by it than others.

Administrator

Some staff members shared that they had personal experience with self-harm, which made them more invested in completing the training to better support students. While school therapists have supervision, staff members may complete the training and then return to their lessons without fully realizing how the content has affected them. As such, the module should also prioritize staff well-being:

I just want to say, make the point that, well, I’m not only speaking for myself, but we’re here, obviously, as professionals, but as parents ourselves, I’ve personally had experience of this, so I just wanted to say that we’ll be speaking from both a professional and a personal perspective.

Assistant principal

There was a strong consensus that all staff members, including those in nonteaching roles, would benefit from the e-learning module. Participants emphasized the importance of equipping all staff members with the skills to respond to potential discoveries or disclosures of self-harm. It was noted that some staff members who are often on the periphery of school training would benefit from being included:

...the canteen staff, for example, do have quite a lot of interactions with the kids.

Associate assistant head

I was a catering assistant for 10 years, and you’re part of a school, but you’re not part of a school. You know nothing. You’re not really included. So, I think catering staff would probably benefit a lot, because they will know absolutely nothing. Because you are secluded.

Food technician and teaching assistant

#### Theme 2: Module Design, Structure, and Content

Staff members suggested that the training module include examples of good practice and opportunities for self-reflection through example scenarios of supportive communication with students. Incorporating scenarios would enhance engagement and help staff understand how to respond more confidently to potential real-life situations. Staff members emphasized the need for training videos to include empathetic and realistic safeguarding messages, clearly informing students that any disclosures may need to be shared with others. Videos should address the common hesitation that staff members can feel in challenging situations and encourage them to seek guidance if unsure. In addition, they recommended including sentence starters and response prompts to help those who are uncertain about how to respond to a discovery or disclosure:

Sentence-starters for a staff member who would have no idea what to say and would panic. If they read those and could see them, I think that’s really helpful.

Deputy special educational needs coordinator

They highlighted that visuals and animations are crucial to keep staff engaged, although some pointed out that animations could be distracting, especially when trying to read the on-screen text simultaneously. For imagery, participants advised against stereotypical depictions of self-harm, recommending instead the use of subtle visuals that reflect the hidden or isolating aspects of self-harm. Importantly, they emphasized that images should be “realistic” and “reflect the diversity” of school communities.

Staff members recommended building in self-reflection activities at the beginning and end of the module to help learners gauge the impact of their learning:

Do you know what would be really powerful, is to do it [self-reflection] at the start and the end. In terms of realizing the students’ perspective, in terms of what’s really going on for them...I just think that that’s, better than a certificate, is for me to know that that was worthwhile. That I’ve got it now, or I feel better equipped if I walk into a scenario like that.

Assistant principal

That depends on your own personal circumstances, as well, as to how you feel you can answer the questions. Because some people are more personally affected by it than others. And like you say—sometimes you get detached. You don’t mean to, but you’ve seen a lot, and—No, but you know, it’s true that in your role, if you see it lot—it’s—it does put a different perspective on it.

Administrator

To meet diverse needs, participants suggested giving learners control over presentation formats, including options for subtitles, voice-overs, and playback features. Videos should ideally be approximately 2 minutes long and interspersed with quiz questions to maintain user engagement. Participants recommended allowing for flexible navigation within the module so that staff members could revisit or skip sections as needed:

Is there an option for you to make a video with no sound and then make a video with sound, and people could choose which one they want. Because I’m dyslexic myself, it’s probably easier for people with learning disabilities to be able to access what you’re talking about if there is sound. But then some people might find that too distracting.

Head of department for art and design

#### Theme 3: Potential Barriers to and Facilitators of Implementation

Staff members supported a whole-school approach that fosters a culture of openness around self-harm. Participants agreed that the training should be provided to all staff members as it would emphasize that safeguarding is a shared responsibility and help create a more supportive school environment:

Really, we all should have knowledge. Because actually, we all have a duty to safeguard. That’s every adult in the college has a duty to safeguard. So, we could provide more benefit and help to students when they’re struggling with other emotional issues...And so, I think every member of staff being trained would help everybody respond...it might just be that we get a better relationship to talking about it, so it’s not such a taboo subject. And then there might be more understanding to help.

Administrator

Participants felt that making the training mandatory and allocating time for its completion would be key to successful implementation. Given busy school schedules, inset training days would be ideal for staff to complete the e-learning module. They recommended that a concise 20- to 30-minute module with the flexibility to start and stop completion would make it more manageable for all staff members:

Time [laughs]. It’s not even that, I don’t know, we could—the whole school, but it would have to be in an inset day, and with that we’d have to find out who the person that runs CPD, if we can have an allocated slot. Because no one’s going to do it in their own time.

Safeguarding officer

While most staff members said that they would expect to complete the training on a desktop, they thought that the training module should be compatible with both desktop and mobile platforms to offer greater choice and flexibility:

It would be good to have the option, like...if you wanted to do it at home, and people had the option to do it on a mobile, it would be useful if it is kind of transferable—I’m sure I looked at it on my phone when I looked at it.

Head of humanities

Ensuring that the training is suitable for catering, administrative, and cleaning staff was seen as important as students may feel more comfortable approaching these individuals. Providing learners with CPD certificates on completion could boost participation and enable senior leaders in the school to track completions and follow up with staff members who have not finished. In terms of scaling up the training, they recommended partnering with reputable educational and mental health organizations and integrating it into teacher education programs:

I did my recent level 3 safeguarding training on a similar module, like this, and it was brilliant, because I could go away, come back, do it, and it worked really, really well.

Assistant principal and teacher

Concerns were raised about the possibility of staff discussions on self-harm with students conflicting with safeguarding protocols, which could potentially hinder schools from adopting the training intervention. To address this, the training should include guidance for staff on responding thoughtfully and nonjudgmentally to disclosures while clearly explaining to students the necessity to share information for their safety.

### Findings From the User Testing

#### Overview

Monitoring questions were embedded into the SORTS module and stored anonymously in a learner record system. First, learners were asked for their “name of school or academy” and could select from a drop-down list of schools drawn from a UK database or “other organization.” Learners were also asked “how would you classify your role?” We generated 3 categories to capture similar roles, responsibilities, and previous training in UK school settings. Participants from the user testing schools self-reported their roles as “teacher, senior leadership or pastoral” (12/20, 60%), “teaching assistant, administrative staff, librarian” (7/20, 35%), and “school facilities (catering, cleaning, technician)” (1/20, 5%). Learners were asked to rate their confidence and understanding of self-harm at the beginning and end of the training. Finally, learners were asked to rate the training. Mean scores are shown in Table 2.

**Table 2:** Participants’ ratings for the monitoring questions embedded into the Supportive Response to Self-Harm e-learning module (N=19)^a^.

Monitoring question	Response scale	Score, mean (SD)	
		Start of module	End of module
“I feel confident that I can effectively handle a conversation with a student about self-harm.”	0—“not at all confident” to 9—“very confident”	5.32 (1.86)	7.47 (1.07)
“I feel confident that I have a good understanding of what causes young people to self-harm.”	0—“not at all confident” to 9—“very confident”	6.05 (1.51)	7.84 (1.01)
“How would you rate this training?”	1—“poor” to 5—“excellent”	—^b^	4.42 (0.77)

^a^Embedded monitoring data did not save for 5% (1/20) of the participants. Therefore, the mean scores presented in this table are based on 95% (19/20) of the user testing participants.

^b^Participants only provided a response for “how would you rate this training?” at the end of the module.

Paired-sample *t* tests (2-tailed) were conducted to compare responses from the start and end of the module. There were significant differences in the scores for the following statements—“I feel confident that I can effectively handle a conversation with a student about self-harm” (t_18_=−7.4717; *P*<.001; equal variances assumed) and “I feel confident that I have a good understanding of what causes young people to self-harm” (t_18_=−7.5607; *P*<.001; equal variances not assumed)—at the start and end of the module.

Tables 3 and 4 show the findings from the usability survey. Results show that there were high ratings for usability metrics, including ease of use and the acceptability of the design and content. Recommendations for improvements were provided in the open-ended survey responses (Table 4) and are discussed in the qualitative themes in the following sections. The key themes from the user testing with staff were (1) usability, (2) content and design, (3) feasibility, and (4) views on how the training improved knowledge and confidence.

**Table 3:** Participant responses to the user testing survey (N=20)^a^.

Survey item			Score, mean (SD)
**Usability**			
	“It was easy for me to access the training module.”	4.76 (0.43)	
	“The objectives of the training module were clear to me.”	4.81 (0.93)	
	“I found the training module easy to use.”	4.71 (0.70)	
	“I needed help to use the SORTS module.”	1.33 (0.89)	
	“I found the videos engaging.”	4.33 (0.78)	
	“I found the quizzes engaging.”	4.05 (0.58)	
	“I liked the design of the module.”	4.29 (0.63)	
	“The images used were relevant to schools.”	4.48 (0.50)	
	“The training content is relevant to my job.”	4.62 (0.58)	
	“I understood the training content.”	4.81 (0.39)	
	“I could easily download and save my CPD certificate.”	4.67 (0.71)	
**Overall satisfaction**			
	“The training was engaging.”	4.38 (0.49)	
	“After completing the training, I feel more confident in how to respond to self-harm.”	4.38 (0.79)	
	“How likely are you to recommend this product to a colleague?”	4.43 (0.58)	
	“The training is important for school staff to complete.”	4.62 (0.49)	
	“I could fit this training into my work schedule.”	4.38 (0.84)	
	“Overall, I was satisfied with the quality of this training module.”	4.62 (0.49)	

^a^All survey items were scored from 1 to 5, with 1=strongly disagree, 2=disagree, 3=neither agree or disagree, 4=agree, and 5=strongly agree.

**Table 4:** Participant feedback on the open-ended usability survey questions (N=20).

Open-ended question	Positive feedback	Suggestions for improvement
“What, if anything, did you not like about the training?”	A total of 14 participants said that there was nothing they disliked.	In total, 7 participants commented on features they did not like: the voice-over was too slow, repeated instructions, and difficult to see on a phone.
“Did you experience anything in the module that would prevent you from completing it?”	All participants said that there was nothing in the module that would prevent them from completing it.	One participant reiterated that they expected the module to be more in depth and show images to help identify self-harm injuries.
“Is there anything that would make you more likely to complete this training?”	A total of 16 participants said that there was nothing that would make them more likely to complete the training and left positive comments, such as describing the module as very informative and succinct.	In total, 5 participants said that they would be more likely to complete the module if there were more videos representing a wider range of self-harm methods, more statistics and information on the causes of self-harm, and allocated CPD^a^ time to complete the module.
“Were there any difficulties for you accessing/beginning the module?”	In total, 20 participants reported no difficulties accessing or beginning the module and described it as very easy, with clear explanations of how the module would be laid out.	One participant thought that their progress would be saved if they exited the module and, therefore, reported having to start it from the beginning after exiting.
“Overall, how would you describe the module?”	A total of 19 participants described the module in very positive terms, including (1) easy-to-digest and accessible entry-level training for all school staff members; (2) very practical, useful, and clear advice; (3) engaging and user-friendly format; (4) soft and relaxing design and colors; (5) information that staff had not encountered in other training; and (6) feeling more confident after completing the module.	—^b^
“If you could change anything about it, what would you change?”	In total, 10 participants said that there was nothing they would change and left positive comments regarding the diversity represented in the visuals and the mixture of interactive features.	Suggestions for changes included myth busting; more challenging quiz questions; further explanation of the scenarios, what went well, and what could be improved; links to additional learning resources throughout the module for those seeking a higher training level; more content representing the student voice; and strategies to put in place beyond the initial conversation.

^a^CPD: continuing professional development.

^b^Not reported.

#### Theme 1: Usability (Ease of Use and Navigability)

Staff generally found the training module easy to start and navigate. They found the structure intuitive and valued the ability to easily return to previous sections:

Really straightforward, and you could just navigate through really easily. I liked how—I think I did something wrong at one point, and I could just really easily navigate back as well. And I liked that because sometimes when you do these trainings, once you’ve skipped forward you can’t go back. And then you think, “Oh no, I didn’t grasp the right thing,” or, “Actually, I want to re-read that.” So, I felt that that was nice, to be able to do that.

Post 16 support officer

However, participants recommended some minor usability improvements to streamline the experience. For example, the “Continue” button was not always visible on certain devices, possibly due to the screen zoom settings, which caused confusion. A couple of participants found it difficult to determine where they were within the module, especially when they returned to the home page without a section being marked as completed. They suggested that a clearer overview at the start could help clarify progress:

I did have a bit where it kind of takes you back out. And it hadn’t said that I’d completed the first module, so I just went back into it. And then I was like, hang on, I’ve done this before. I ended up flicking through the first module again just to check that I had done it before I came, went into the second one. So maybe just like a tick screen or something [showing] that that’s completed.

Librarian

#### Theme 2: Content and Design

Participants generally felt that the training module provided an appropriate level of information, with content that was easy to understand and well balanced for both teaching and nonteaching staff. However, some information was repeated multiple times, which some users found frustrating. Overall, they found the module easy to follow as information was presented in manageable “bite-sized pieces” that were not overwhelming:

I felt like it was really digestible chunks of information. I never felt overwhelmed by it. And I think sometimes, especially with that content—and I think that’s one of the reasons I wanted to do it, so I didn’t feel that overwhelm. But I never did. I felt like I could follow it really nicely through.

Head of geography

Staff members with previous mental health training said that the training content reinforced their existing knowledge and validated their instincts on how to approach sensitive situations and handle difficult conversations with students:

The thing I found the most useful from it was there were some bits where I was going, “OK, I thought that would have been the approach I would have taken” on a scenario...So it wasn’t for me going, “Oh, I know this.” It was actually gaining that confidence of going, “Actually, what my instincts were saying were down the right lines.” I think that would be helpful for everyone in that way.

Head of drama

All staff members valued the “knowledge check” and “self-reflection” questions, which helped reinforce understanding of key facts and gauge their understanding and progress throughout the module. They found that the self-reflection prompts specifically encouraged them to consider their own feelings and responses in real-life scenarios:

I really liked the one with the timer...because it wasn’t too long, and it just made me sit there for 20 seconds and go, “OK, how would I actually feel?” and actually think about it. Not for so long that it felt like a drag. But for just a little bit of time to have something slightly more than a surface-level thought.

Assistant principal

Yeah, I quite like the fact that there were two different types. And I like that it’s kind of integrated throughout the modules as well, because I find that, well, it’s all saved up towards the end. If I get something wrong, I don’t always go back and double check what I was supposed to have. And it’s nice that it’s kind of right there, so that it’s you’ve just read it through, and then you kind of have the reconfirmation of the facts as you’ve completed the questions as well. And then the more open-ended ones I did. I quite like those, because it was like, well, it made you really, actually consider what would happen in a real-life scenario. So that was good too.

Librarian

Participants found the module visually engaging, particularly the filmed scenarios, which they said were realistic and inclusive. While some initially worried that one of the videos depicted an incorrect approach, they soon realized that this was intentional and illustrated a progression to best practice. This highlighted the need to provide additional information before each video scenario.

In addition, they appreciated the inclusion of a male student in one of the videos as it helped challenge the stereotype that self-harm is primarily a female issue. This was seen as a constructive way to break down gender stereotypes around mental health, making the module more inclusive and relatable to a broader audience:

I really liked that the video was a boy. I think that made it so that—I feel like sometimes the stereotype is that it’s females who will cut themselves, not necessarily different types of self-harm. And I thought it was really good that it was a man speaking to a boy. It just jumped over that hurdle, that barrier or the stereotype that we have.

Social sciences teacher

Quite a few of us are support staff. I would have quite liked something that was not classroom teacher-based, because the situations in which we interact with students are not the same.

Assistant principal

#### Theme 3: Feasibility (Implementation and Adoption)

There was a consensus that the module’s flexible format would make it easy to implement in schools. The 30-minute duration was considered ideal as it would allow staff members to gain essential insights without a major time commitment. Senior staff members said that they would incorporate the module into allocated training time, such as staff inset days and CPD sessions or as part of new staff induction programs:

I think I prefer doing it as a whole-staff CPD, not in one go. Because poor people, when they start at schools, it’s information...it can’t go on forever, because you won’t get the buy-in from schools. But I think to go, “Right, this is going to take you 20 minutes guys, and it could be really helpful,” would be how I see it being done.

Assistant principal

One senior staff member suggested that staff members could complete the training as a group rather than individually. Participants highlighted the benefits of allowing time for discussions during or after the training to reinforce learning and facilitate self-reflection. They acknowledged that the topic could be distressing for some staff members and support should be available immediately after training to address any issues that arise. This highlighted the need for clear implementation guidelines for schools using the training.

#### Theme 4: Views on How the Training Improved Knowledge and Confidence

The training was valued for fostering a shared understanding among staff members and establishing a consistent “baseline” for approaching self-harm within the school. Staff members appreciated how the module explained the complex and personal nature of self-harm, highlighting that it may not always have a clear cause. The training broadened their awareness of the different forms that self-harm can take beyond visible injuries, helping them recognize that less obvious behaviors, such as ingesting harmful substances, can also be forms of self-harm. This increased their understanding and awareness of different warning signs:

I think it was really beneficial having the different types of self-harm, as we automatically just think of cutting, and obviously there’s so many. So I think that was really good to have a dedicated section on that.

Special educational needs coordinator

Things like hair-pulling and all those things that are maybe less easy to pick up on. And in general, it’s an incredibly secretive thing anyway, so people usually go to great pains to keep it to themselves.

Designated mental health lead

Some participants already felt knowledgeable about self-harm due to their personal experiences or from interactions with students who were struggling with self-harm. For these individuals, the training provided reassurance that they were handling disclosures appropriately and offering a supportive response. Others noted that this training would be particularly beneficial for nonteaching staff members who do not work as closely with students, as it would provide them with essential insights and help them notice the warning signs:

I would feel really good about other people having done the same knowledge, and knowing that this is the level that other people—that everyone in the school has had...So that if I had to refer it on to someone—I know that they’d be getting this kind of positive approach.

Assistant head

Overall, participants said that the training improved their confidence in recognizing and responding to self-harm, and it emphasized the importance of a compassionate approach. Staff with previous mental health training felt that the e-learning module reinforced their confidence in addressing self-harm and affirmed their ability to approach situations appropriately. They particularly liked the training’s emphasis on maintaining a supportive, conversational approach rather than focusing solely on procedural aspects. However, some suggested including guidance on handling situations in which students are unwilling to talk:

For me, that was my first training I’ve done on the mental health. So I was going into it not really knowing too much. But after, I felt a lot more confident of signs, what to do. So yeah, it made me feel like, OK, if someone comes to me, I knew how to approach it and go about it.

Administrator

Several staff members found it helpful that the training highlighted the importance of positive body language and eye contact, which they felt would build trust and make students feel genuinely heard. One participant suggested that body language tips may need to be adapted for students with autism or attention-deficit/hyperactivity disorder, and this led to a discussion on the need for an additional training module specifically addressing self-harm among neurodiverse students:

I really, really, really agree with the whole you have to communicate non-verbally. But I will say on the body language and eye contact thing, as someone who works almost exclusively with neurodiverse children, sometimes the things that are good advice if you’re talking to a neurotypical child, would come across very intimidating and demanding to a neurodiverse child.

Higher-level teaching assistant

## Discussion

### Summary of Findings

This manuscript outlines the collaborative design and development of a self-harm awareness e-learning module for school staff. We also present the qualitative and quantitative evidence of the module’s usability and acceptability from a diverse sample of school staff members, including those in senior leadership, teaching, administrative, and school catering roles. The training module builds on previous work [23,25] and addresses the lack of effective and acceptable training to support whole-school awareness of self-harm [17,18]. We identified one recent study that developed an e-learning module on self-harm for schools; however, it was specifically designed for teachers rather than a wider range of teaching or nonteaching staff, which does not fit with a whole-school approach. In addition, the study provided limited details on the training content and development process and did not appear to use a user-centered or person-based approach. Moreover, the absence of follow-up data makes it challenging to assess the long-term impacts of the training [21].

In contrast, the SORTS e-learning module follows a whole-school approach in alignment with the UK government’s national policy to improve mental health prevention and intervention for children and young people in educational settings [8]. It recognizes that all school staff members are uniquely positioned to intervene when they identify a young person who has self-harmed, helping prevent further escalation and guiding students toward appropriate support. Research has shown that school staff are often the first point of contact for parents concerned about their children’s well-being, with schools being described as a “one-stop-shop” for advice for parents before seeking help from health professionals [25,35,36]. To support a whole-school approach, it is essential that all school staff members receive the necessary training and guidance to effectively respond to students who self-harm [37].

In this study, feedback from the 2 participatory design cycles was instrumental in shaping the design and development of the SORTS e-learning module. We involved relevant users throughout the development process, a key step in improving the acceptability and uptake of interventions [38,39]. Consistent with previous research, most school staff members reported feeling ill-equipped and stated that they had not received self-harm awareness training, highlighting an unmet need for such training [11,17,18,40]. Some participants noted that their awareness and understanding of self-harm were shaped by their previous personal and professional experiences, resulting in considerable variation in staff knowledge within schools. The co-design workshops gathered valuable staff input on the content, design, and structure of the module before prototype development. Several user requirements were identified, including a simple, user-friendly interface; accessibility features; interactive elements to engage users; and concise information to avoid overwhelming staff. Participants also requested model responses in video format and suggested including sentence starters for situations in which they felt unsure of what to say. In addition, they highlighted the importance of opportunities for reflection to explore their own beliefs and experiences related to self-harm. This aligns with evidence that reflection is a key mechanism supporting effective implementation of interventions in school settings [41]. In response, we created 2 video scenarios demonstrating best practices with suggested sentence starters to support staff in navigating difficult conversations regarding discovery or disclosure. We included references to the school’s safeguarding protocols to reinforce safe, effective procedures. For accessibility, a voice-over option was added, and the module was designed to function seamlessly across different devices. To enhance usability, we ensured that the development was based on the general principles of interaction design [42].

User testing showed that the training was easy to use and navigate, with both the content and design being highly acceptable to staff. Results showed an increase in staff’s knowledge and confidence, and user testing focus groups indicated that this was particularly true for those with no previous mental health training. This is important because previous research has found that a lack of knowledge and confidence can lead to negative responses [13,40]. For those with previous mental health training, the SORTS module reinforced their existing knowledge and provided reassurance that they were responding supportively to students. For many participants, the module helped them recognize a wider range of self-harm behaviors, suggesting that, without training, school staff may overlook certain behaviors or fail to respond effectively due to a lack of awareness.

Stigma is a major barrier to help seeking. Research indicates that young people are often reluctant to disclose their self-harm due to concerns about receiving a negative response [14,16]. However, supportive student-teacher relationships, characterized by warmth and understanding, play a crucial role in promoting student well-being [25]. Therefore, training must be integrated into a broader cultural shift that fosters a whole-school approach, which includes implementing a school policy on self-harm; incorporating relevant education into the curriculum; and providing support for parents, siblings, and peers of students who self-harm [23,37].

### Strengths and Limitations

A key strength of this study is the rigorous co-design methodology. It was conducted in line with Medical Research Council guidelines for complex interventions, involving regular input from “appropriate users” at all stages of intervention development to ensure that the training intervention is acceptable, feasible, and engaging [43]. It applied a person-based approach to guide the development [26]. Staff workshops ensured that school staff members’ perspectives were incorporated throughout the iterative design process. Another strength of this study is the mix of urban and rural schools with diverse student populations that participated in the study. Schools were in regions where the local population has limited opportunity to take part in health research. We recruited school staff members in a range of roles to ensure a range of views from teaching and nonteaching staff.

Despite the key strengths outlined previously, some limitations should be acknowledged. Those who volunteered to participate in this study are more likely to have an interest in student mental health, and therefore, we may not have captured the views of staff members who are more concerned about responding to a student who is self-harming. Some decisions about the e-learning module content and design were made before the staff workshops as it was necessary to have content for staff to consult on. However, this previous work was developed in consultation with experts in mental health, education, and specifically self-harm. A limitation of this study could have been that young people were not involved in the development work. However, we have previously conducted 2 separate studies with young people about training school staff about self-harm, which directed the research in this study [23,25].

### Implications and Future Work

The SORTS e-learning module was designed for all school staff members to support a whole-school approach to self-harm prevention and intervention. We anticipate that implementing this approach in schools will encourage young people to seek help from staff members, improve signposting, and facilitate access to community resources and support. While it is targeted at school staff, it may also be relevant for other professionals working with young people, for example, those in youth organizations, social care, or juvenile justice.

The module is already being used by schools, and preliminary data collected before and after training suggest that staff knowledge and confidence increase after completing the training-learning module. We are now planning a feasibility study to evaluate the module’s effectiveness across several schools, assessing both staff and student outcomes.

In addition, staff members in this study highlighted gaps in their training related to other areas of mental health. Future work could involve co-designing similar modules on topics such as absenteeism, eating disorders, and supporting neurodiverse students.

## Appendix

Multimedia Appendix 1 Supportive Response to Self-Harm theory of change model.

Multimedia Appendix 2 Co-design workshop topic guide.

Multimedia Appendix 3 User testing survey.

Multimedia Appendix 4 User testing topic guide.

## Abbreviations

COREQ: Consolidated Criteria for Reporting Qualitative Research
CPD: continuing professional development
SORTS: Supportive Response to Self-Harm

## Data Availability

The datasets generated or analyzed during this study are available from the corresponding author on reasonable request.
