# Supplementary material for: A Self-Harm Awareness Training Module for School Staff: Co-Design and User Testing Study
Source: JMIR Form Res. 2025 Jun 2;9:e69309. doi: 10.2196/69309 (PMC12171642; doi:10.2196/69309)
Supplement: Multimedia Appendix 1 [file formative_v9i1e69309_app1.pdf]

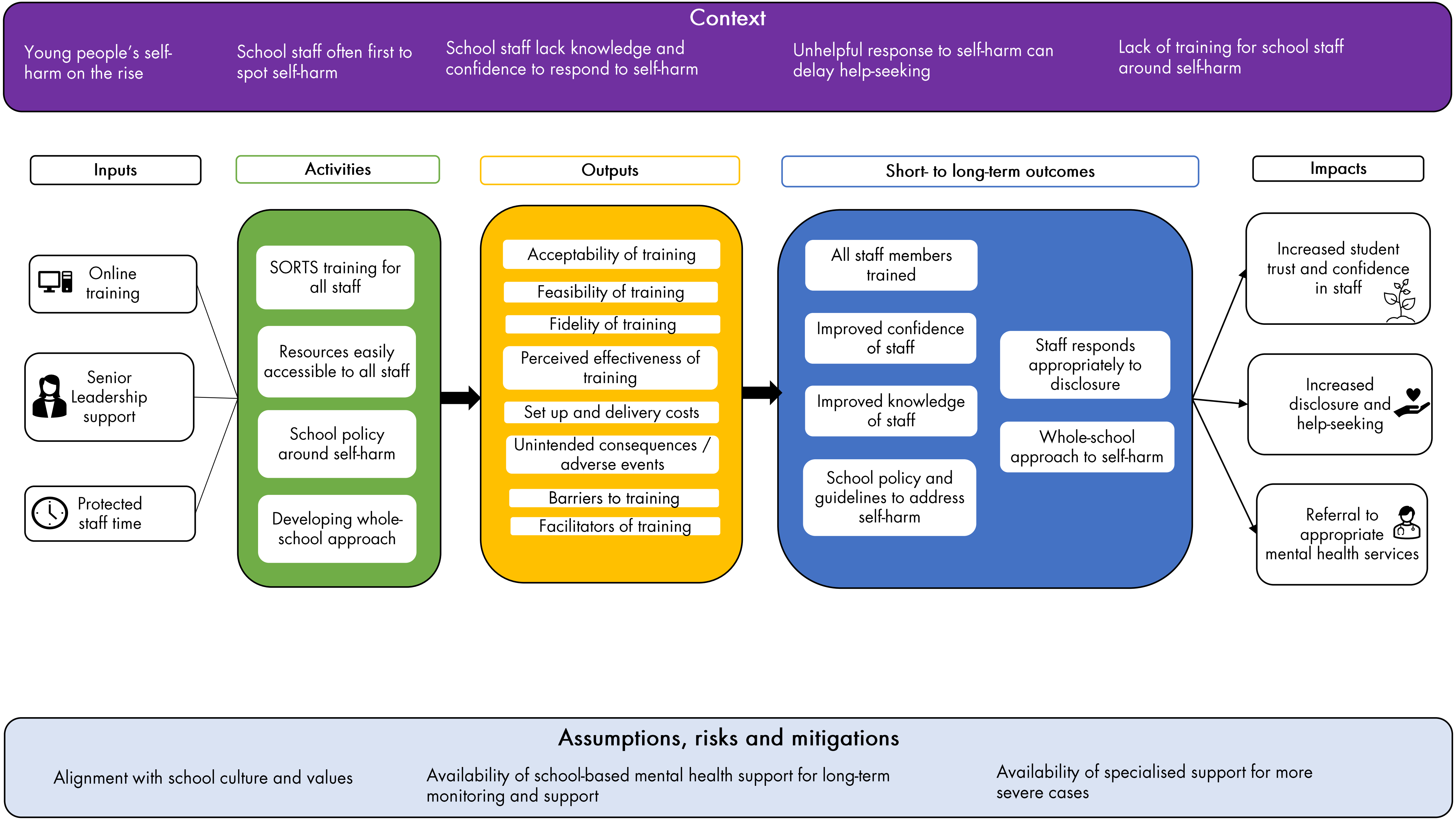
Context
Young people's self-harm on the rise
School staff often first to spot self-harm
School staff lack knowledge and confidence to respond to self-harm
Unhelpful response to self-harm can delay help-seeking
Lack of training for school staff around self-harm
Inputs
Online training
Senior Leadership support
Protected staff time
Activities
SORTS training for all staff
Resources easily accessible to all staff
School policy around self-harm
Developing whole-school approach
Outputs
Acceptability of training
Feasibility of training
Fidelity of training
Perceived effectiveness of training
Set up and delivery costs
Unintended consequences / adverse events
Barriers to training
Facilitators of training
Short- to long-term outcomes
All staff members trained
Improved confidence of staff
Improved knowledge of staff
School policy and guidelines to address self-harm
Staff responds appropriately to disclosure
Whole-school approach to self-harm
Impacts
Increased student trust and confidence in staff
Increased disclosure and help-seeking
Referral to appropriate mental health services
Assumptions, risks and mitigations
Alignment with school culture and values
Availability of school-based mental health support for long-term monitoring and support
Availability of specialised support for more severe cases
