# Supplementary material for: A Self-Harm Awareness Training Module for School Staff: Co-Design and User Testing Study
Source: JMIR Form Res. 2025 Jun 2;9:e69309. doi: 10.2196/69309 (PMC12171642; doi:10.2196/69309)
Supplement: Multimedia Appendix 2 [file formative_v9i1e69309_app2.docx]

Workshops Facilitator Guide

**Confirmation of Informed Consent**

- Confirm that every participant has had an opportunity to ask questions and sign the consent form
- Answer any outstanding questions

**Ground Rules**

- Listen to and respect each other’s perspectives.
- Keep the information shared confidential.
- Discussion is being recorded for transcription purposes, but we ask that participants do not record, take photo or video during the workshop.
- Do not interrupt each other.
- Participants are welcome to leave to attend to their needs.
- If participants do not want to verbally share an idea, they may write it on their feedback form or supplied paper.
- [Additional ground rule suggestions from participants.]

**Icebreaker**

- Name introductions
- Where are you from?
- What brought you to the workshop today?

*NB The guide gives an indication of the type and range of questions which will be covered in the workshop. The discussion will develop in response to the participant’s contribution, and we will pick up and explore issues of particular relevance and salience to each aim. The wording and direction of questions will be tailored to individual participant’s circumstances and phrased sensitively and appropriately according to context.*

| Topic/aim | Materials | Activity | Prompts |
| --- | --- | --- | --- |
| Refine and finalise the training scenarios. | - Two storyboards with script (one for each of the proposed videos) - Orange and green sticky notes | Ask participants to write notes on the storyboards. Green sticky notes for useful/well-liked parts of the script/scenes and orange for things they think could be problematic or they have a query on.  Researchers will facilitate discussion around responses. | *Do you find the scenarios relatable?*  *“…” realistic?*  *What three words would you use to describe each scenario?*  *Are there any parts of the script that you find particularly useful?*  *Are there parts of the script that you disagree with?* |
| Refine and finalise UI design | - Pre workshop feedback form - Print out/whiteboard | Ask participants to mark on a 0-100 scale. Researchers will facilitate discussion around responses. | *How much do you agree that the design looks current?*  *“…” relevant to schools?*  *“…” appropriate for the topic?*  *“…” professional?* |
| Capture potential users views on prospective acceptability | - Pre workshop feedback form | Ask participants to share notes from their pre-workshop feedback form on features that improve acceptability and engagement (quizzes, knowledge checks, animation, narration, multiple choice, length of the modules). | *Did you feel there was anything missing from the overview of the e-learning module?*  *Did you feel there was anything that could be taken out from the overview of the e-learning module?*  *What were your first impressions of the aesthetics?*  *How did you find the quiz? Was it informative, engaging, surprising, too easy, too hard?* |
| Capture potential users views on prospective feasibility | - Whiteboard | Researchers will lead a discussion on the barriers and facilitators to implementing the training module in school settings and write these into a table. We will ask participants to add their suggestions of solutions to the barriers, or ways to ensure the facilitators are in place. | *How feasible do you think it would be to complete this training module at work?*  *Can you think of any barriers to you completing this training?*  *How easy would it be to share the module with others?*  *Is there anything that would make it more likely that you would complete the training module?* |
