# Supplementary material for: A Self-Harm Awareness Training Module for School Staff: Co-Design and User Testing Study
Source: JMIR Form Res. 2025 Jun 2;9:e69309. doi: 10.2196/69309 (PMC12171642; doi:10.2196/69309)
Supplement: Multimedia Appendix 3 [file formative_v9i1e69309_app3.docx]

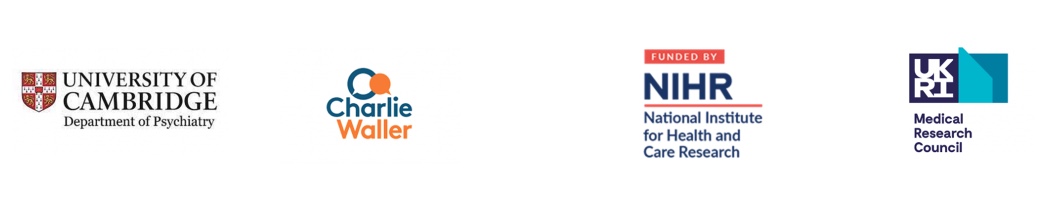


**SORTS project** (Supportive Response to Self-Harm in Schools)

**Feedback on Training Module**

Thank you very much for completing the SORTS training module.

We would really like to know what you think about it . Please complete this survey, your responses will help us to make improvements to the module.

Please enter your name so we can keep track of those who have completed the survey. Your answers will be anonymous and confidential.

If you have any questions, please contact Hayley [add email]

………………………………………………………………………………………

**Please read each statement and indicate the extent to which you agree or disagree from 1 to 5, where 1 = strongly disagree, 2 = disagree, 3 = neither agree or disagree, 4 = agree, 5 = strongly agree.**

It was easy for me to access the training module.

The objectives of the training module were clear to me.

I found the training module easy to use

I needed help to use the SORTS module.

I found the videos engaging.

I found the quizzes engaging.

I liked the design of the module

The images used were relevant to schools.

The training content is relevant to my job.

I understood the training content.

I could easily download and save my CPD certificate.

**Overall satisfaction**

The training was engaging

After completing the training, I feel more confident in how to respond to self-harm.

How likely are you to recommend this product to a colleague?

On a scale from 1 to 5 (1=not at all likely, 5=very likely):

The training is important for school staff to complete. (agree to disagree)

I could fit this training into my work schedule.

Overall, I was satisfied with the quality of this training module. (satisfaction scale)

**Open ended comments**

What, if anything, did you not like about the training?

Did you experience anything in the module that would prevent you from completing it?

Is there anything that would make you more likely to complete this training?

Were there any difficulties for you accessing/beginning the module?

Overall, how would you describe the module?

If you could change anything about it, what would you change?
