# Supplementary material for: A Self-Harm Awareness Training Module for School Staff: Co-Design and User Testing Study
Source: JMIR Form Res. 2025 Jun 2;9:e69309. doi: 10.2196/69309 (PMC12171642; doi:10.2196/69309)
Supplement: Multimedia Appendix 4 [file formative_v9i1e69309_app4.docx]

**User Testing Facilitator Guide**

**Introduction:**

- Thank the participant for attending.
- Discussion is being recorded for transcription purposes.
- Confirm that the participant has had an opportunity to ask any questions.
- If participants do not want to verbally share an idea, or think of something after the session, they also have the option to get in touch via email afterwards.

**For the first half-hour, we’ll ask for your thoughts about the training module and how it can be improved. In the second half, we’ll discuss more general questions about whether the training has improved your knowledge and confidence in addressing the issue.**

1. **Usability and Acceptability:**

**Objectives:**

- when you first started the training, how clear were the objectives to you?
  - If the objectives weren’t clear, how did that impact your experience or feelings about the training?
  - What suggestions do you have for making the objectives clearer?

**E-Learning Module Level:**

- How clear was it to you that the module was designed as entry-level training for all school staff, including admin, non-teaching and teaching roles.
- Would you prefer more clarity about who the module is aimed at?

**Usability:**

- How easy or difficult was it for you to start the module?
- Would it help to add some guidance?
- Why was it easy to access?
- Is it similar to other types of training previously completed?
- Did anyone have any problems or difficulties with the module?
- Were there any issues about exiting the module and it not saving? - Mention GDPR and privacy.
- Would a skip button on the monitoring questions help?

**Complexity:**

- Do you feel the module was pitched at the right level for training aimed at all staff, including both teaching and non-teaching roles?
  - Easy to understand?
  - Would you prefer more in information e.g., adding some research basis for points?
  - Follow up module with more information

**Videos:**

- What did you think about the videos?
  - engaging?

**Quizzes:**

- Explain there were knowledge test questions and self-reflection questions:
- Were they helpful in reflecting on your knowledge? Why?

**Feasibility:**

- How would this practically work within your school?
- Is the time okay?
- Would it fit into your schedule?

1. **Impact and utility of training**

**Psychological Effects:**

**Confidence:**

- How did your confidence in responding to self-harm change before and after completing the training?
- Did the module help you feel more confident? How?
- Do you have any concerns about discussing self-harm with a student?

**Understanding**

- How did completing the module impact your understanding of self-harm, and were there specific areas where you felt your knowledge increased?

**Whole School Approach:**

- This training has taken a whole-school approach to self-harm. Do you think all staff should be required to complete this training, or should they have the option to opt out if they feel uncomfortable?

**Summary Questions:**

- Would you be interested in additional training on self-harm, such as more advanced or specialised courses?
- What about information or resources do you think would be helpful for young people and parents regarding self-harm?
- Any there any other areas of mental health where you would like to receive more training?
